# Identification, Purification and Characterization of Laterosporulin, a Novel Bacteriocin Produced by *Brevibacillus* sp. Strain GI-9

**DOI:** 10.1371/journal.pone.0031498

**Published:** 2012-03-05

**Authors:** Pradip Kumar Singh, Chittpurna, Ashish, Vikas Sharma, Prabhu B. Patil, Suresh Korpole

**Affiliations:** Council of Scientific and Industrial Research-Institute of Microbial Technology, Chandigarh, India; Auburn University, United States of America

## Abstract

**Background:**

Bacteriocins are antimicrobial peptides that are produced by bacteria as a defense mechanism in complex environments. Identification and characterization of novel bacteriocins in novel strains of bacteria is one of the important fields in bacteriology.

**Methodology/Findings:**

The strain GI-9 was identified as *Brevibacillus* sp. by 16 S rRNA gene sequence analysis. The bacteriocin produced by strain GI-9, namely, laterosporulin was purified from supernatant of the culture grown under optimal conditions using hydrophobic interaction chromatography and reverse-phase HPLC. The bacteriocin was active against a wide range of Gram-positive and Gram-negative bacteria. MALDI-TOF experiments determined the precise molecular mass of the peptide to be of 5.6 kDa and N-terminal sequencing of the thermo-stable peptide revealed low similarity with existing antimicrobial peptides. The putative open reading frame (ORF) encoding laterosporulin and its surrounding genomic region was fished out from the draft genome sequence of GI-9. Sequence analysis of the putative bacteriocin gene did not show significant similarity to any reported bacteriocin producing genes in database.

**Conclusions:**

We have identified a bacteriocin producing strain GI-9, belonging to the genus *Brevibacillus* sp. Biochemical and genomic characterization of laterosporulin suggests it as a novel bacteriocin with broad spectrum antibacterial activity.

## Introduction

Bacteriocins are ribosomally synthesized antimicrobial peptides and have drawn attention in recent years due to their potential therapeutic applications in treating bacteria, including multiple drug resistant bacteria [1]–[3]. Bacteriocins from lactic acid bacteria have been in use for a while as natural preservatives in food industry [4], [5]. Although bacteriocins were originally found to be produced by *Lactobacillus* only, it was subsequently shown to be produced by different species and multiple strains. Consistent with this, many species belonging to the genus *Bacillus* as well as other Gram-positive and Gram-negative bacteria were shown to produce bacteriocins and/or bacteriocin-like substances [6]. In fact, it is observed that majority lineages of bacteria are shown to produce at least one bacteriocin as part of their defense mechanism [7], [8]. Likewise many species belonging to the genus *Bacillus* were reported to produce bacteriocins and/or bacteriocin like substances [9]–[11].

Antimicrobial peptides produced by bacteria are categorized into different classes based on structural and functional characteristics [12]. The class I bacteriocins called as lantibiotics, are well studied with wide applications in both therapeutic and preservation of food products at industrial scale [1], [13], [14]. Class II bacteriocins are further divided into different sub classes, including antilisterial one-peptide pediocin like bacteriocin as sub-class IIa [15]–[18], the two-peptide bacteriocins as sub-class IIb [14], [17]–[19], sub-class IIc containing cyclic bacteriocins and sub-class IId composed of one-peptide non-cyclic bacteriocins that show no sequence similarity to other bacteriocins [14], [17], [20]. Among these different classes of bacteriocins, lantibiotics are found to undergo post translational modifications such as dehydration of Ser and Thr to produce dehydroalanine (Dha) and dehydrobutyrine (Dhb) respectively. The lantibiotics are further divided into two groups based on their structure as group A contains globular lantibioitics and group B composed of linear lantibiotics. Both groups were also found to differ in their mechanism of inhibition activity. Most of the bacteriocins produced by *Bacillus* spp. belong to the class I lantibiotics and exhibit broad range molecular mass, from smallest lichenin (1.3 kDa) produced by *B. licheniformis* [21] to largest thuricin (950 kDa) produced by *B. thuringiensis* [22]. Moreover, they were found to be active at relatively high temperatures and over a wide pH range [23] with potential for therapeutic as well as industrial applications. For example, mersacidin is one such bacteriocin produced by *Bacillus* sp., which inhibits the growth of methicillin resistant *Staphylococcus aureus* [24].

While several species belonging to genera *Bacillus* and *Paenibacilus* are shown to produce different bacteriocins, the antimicrobial substances produced by few *Brevibacillus* taxa have not been characterized in detail. Furthermore, *B. brevis* [25], [26] and *B. laterosporus* [27] have been shown to produce bacteriocin-like inhibitory substances (BLIS) with wide pH and temperature stability. Other than these, *B. laterosporus* strain SA14 was shown to produce two antibiotics [28]. In the present study, we describe the isolation, purification and characterization of a bacteriocin produced by *Brevibacillus* sp. strain GI-9 exhibiting broad spectrum antibacterial activity.

## Results

### Phenotypic characterization

Most of the strains isolated from subsurface soil sample with antimicrobial activity were found to be facultative anaerobes (see Methods). Among these, a strain designated as GI-9, exhibited broad spectrum antibacterial activity by inhibiting the growth of *B. subtilis* (MTCC 121), *S. aureus* (MTCC 1430), *E. coli* (MTCC 1610), *P. aeruginosa* (MTCC 1934) and *L. monocytogenes* (MTCC 839). Phenotypic properties and 16 S rRNA gene sequence (EMBL accession No. FR686596) BLAST analysis of the strain GI-9 assigned it to the genus *Brevibacillus*. It had high percent identity (99%) of the strain GI-9 16 S rRNA gene to that of *B. laterosporus* DSM 25 and showed less than 97% with other species of the genus. However, it showed differences in phenotypic properties such as growth at 50°C, positive reaction for Voges Proskauer test and urea hydrolysis compared to *B. laterosporus* DSM 25.

### Antibacterial activity assay

**Figure 1:**
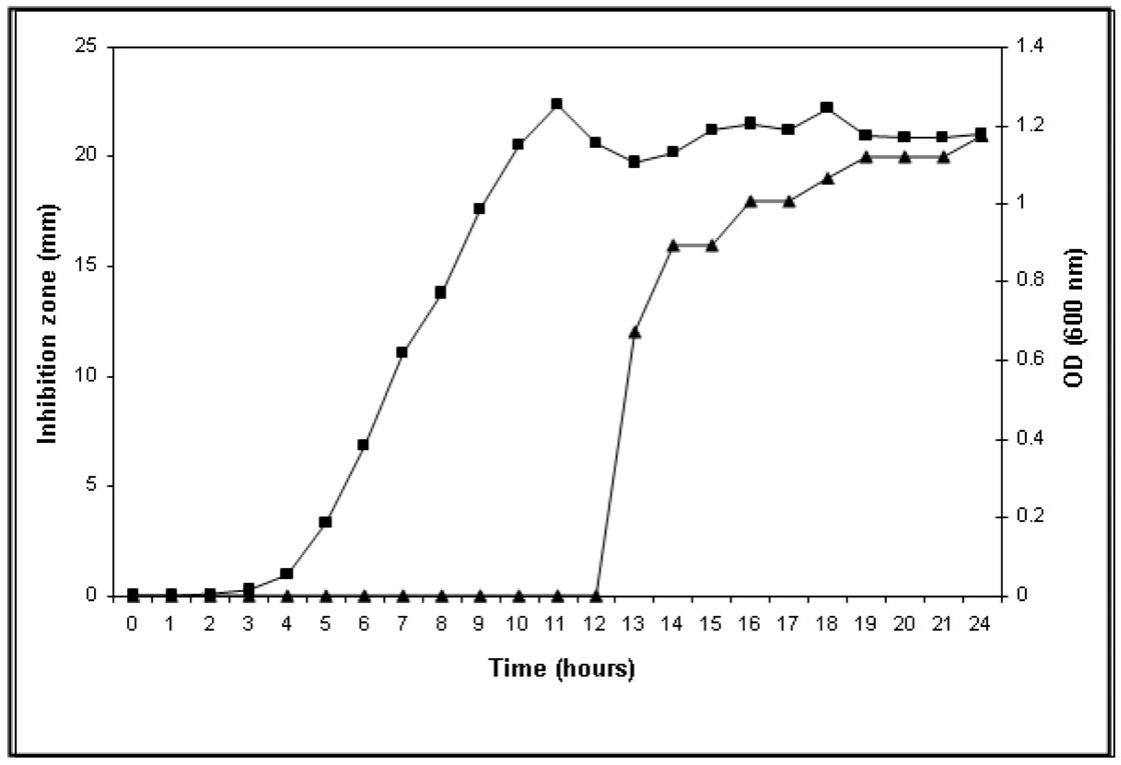
Comparison of bacteriocin production at different phases of growth curve. The growth curve analysis of strain *Brevibacillus* sp. GI-9 indicates that the production of bacteriocin initiated at late logarithmic phase. Growth measured as absorption at 600 nm is indicated by squares (left y axes), while the bacteriocin activity by triangles (right y axes).

Cell-free fermented broth (CFB) collected at indicated time intervals during growth of strain GI-9 were used to perform antimicrobial activity assays. The results showed that bacteriocin production initiated after 12 hours of lag phase. However, there was a significant increase in bacteriocin production between 13 to 18 hours (**Fig. 1**) and the antimicrobial activity remained constant thereafter as measured by inhibition zone. Although it displayed a broad-spectrum antibacterial activity, it did not show any growth inhibition against yeast or fungi. Notably, the medium composition did not influence antimicrobial activity of the bacteriocin as similar results were obtained when GI-9 was grown in minimal medium and subsequently assayed for antimicrobial activity. The growth inhibition studies showed that test strains *B. subtilis* and *S. aureus* were more sensitive than other strains.

### Purification of laterosporulin

**Figure 2:**
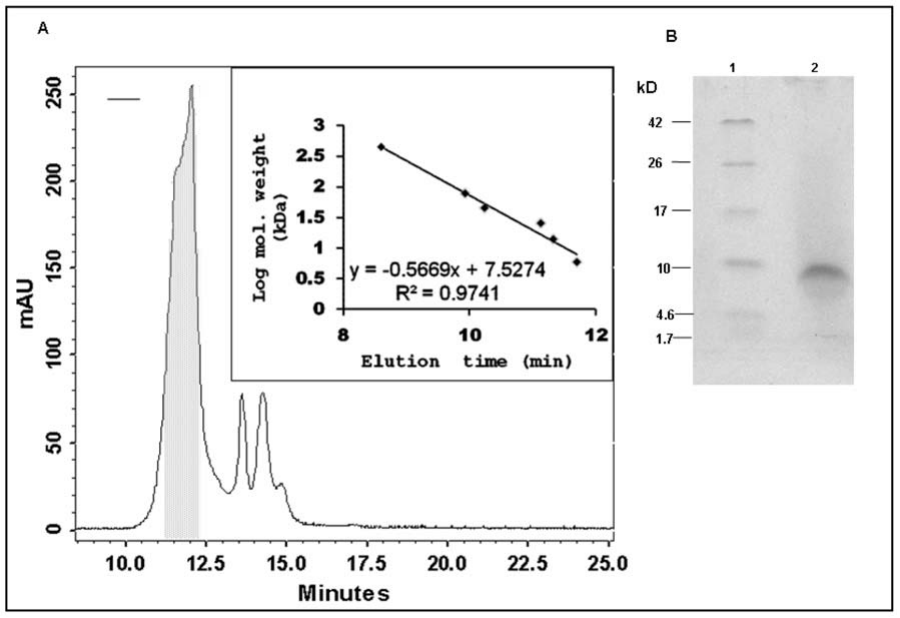
Gel filtration elution profile of laterosporulin (A) and analysis (B). (A) The major peak (highlighted fraction) in gel filtration chromatography shows antimicrobial activity and the calibration curve obtained from protein standards indicates mass of the peptide in the range of 5.3 to 10.1 kDa. (B) Tricine-SDS-PAGE analysis of the major peak showed a single band; lane 1, molecular weight markers; lane 2, purified laterosporulin.

The CFB obtained after 48 hours growth was tested for antimicrobial activity. The bacteriocin present in CFB was extracted by affinity chromatography using Diaion HP-20 and purified by a combination of chromatographic techniques. To remove large proteins, the crude extract was passed through a 30 kDa protein concentrator. The filtrate obtained was found to exhibit antimicrobial activity and the same was applied on to gel filtration columns for purification. The predominant peak shown in **Fig. 2A** exhibited antimicrobial activity on test strains. Re-injection of this predominant peak gave only one peak at the same retention time suggesting that the minor peaks observed in the previous chromatogram were not degradation products of the antimicrobial peptide. Based on the elution profile of standards, the peak with antimicrobial activity may have mass in the range of 5.3 to 10.1 kDa. This peptide was finally purified on a semi-preparative reversed-phase-HPLC. The purified peptide showed a single peak at UV absorption of 220 nm and was positive for antimicrobial activity. Tricine-SDS-PAGE analysis of this peptide yielded a single band (**Fig. 2B**) and the same was used for molecular mass and N-terminal sequencing.

### Determination of molecular mass and N-terminal sequence analysis

**Figure 3:**
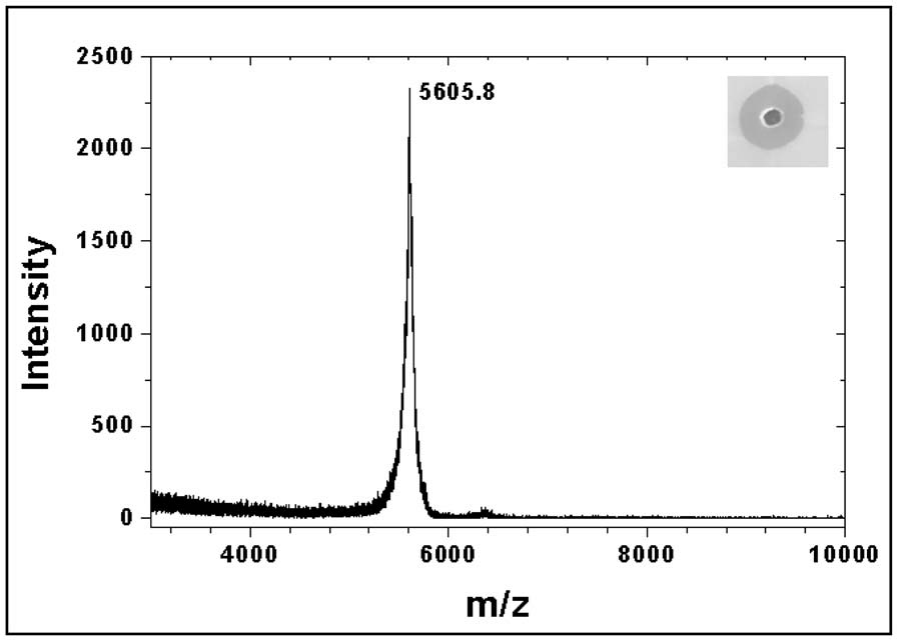
MALDI-TOF mass spectrometry analysis of laterosporulin. The purified bacteriocin from *Brevibacillus* sp. GI-9 shows the mass (m/z) of 5.6 kDa.

The molecular mass observed for laterosporulin by MALDI-TOF was 5605.8 Da (**Fig. 3**), which is in good agreement with mass obtained by gel filtration chromatography (**Fig. 2A**). The N-terminal sequence of laterosporulin yielded partial sequence of ^1^A(M)Q(C)QG(C/Q)PDAISGWTHTDYQCH^19^ for two independently processed samples. The uncertainties at the 1^st^, 2^nd^ and 4^th^ positions obtained in N-terminal sequences were considered to predict the antibacterial activity of each possible peptide sequence using a database of peptides with known antimicrobial potency. Notably, one of the peptides (ACQCPDAISGWTHTDYQCH) predicted highest relative activity compared to other combinations. To confirm the antimicrobial activity of this partial peptide, it was custom synthesized, mass was confirmed by MALDI-TOF and the synthetic peptide was examined in growth inhibition assays using indicator strains *B. subtilis* and *E. coli*. The peptide exhibited weak antimicrobial activity at significantly higher concentration. Upon extensive bioinformatic analysis, no significant similarity was observed to this partial sequence with available bacteriocins in the databases like BACTIBASE or Antimicrobial Peptide database. Also, careful inspection of peptide mass fingerprinting (PMF) data using the databases such as MASCOT and PROWL revealed no similarity with any antimicrobial peptides.

### Minimum inhibitory concentration analysis

**Figure 4:**
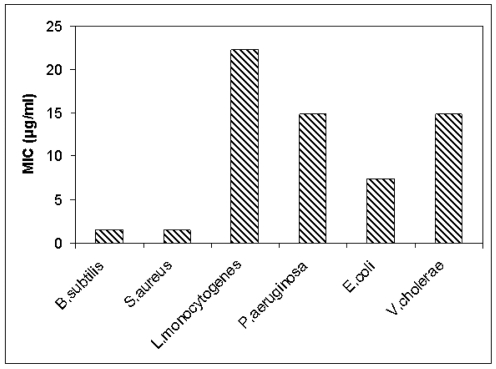
Determination of MIC for laterosporulin produced by strain GI-9. The MIC assay for Gram-positive and Gram-negative bacteria with purified laterosporulin using micro-titer plates in triplicates revealed that *B.subtilis* and *S. aureus* are highly sensitive.

**Figure 5:**
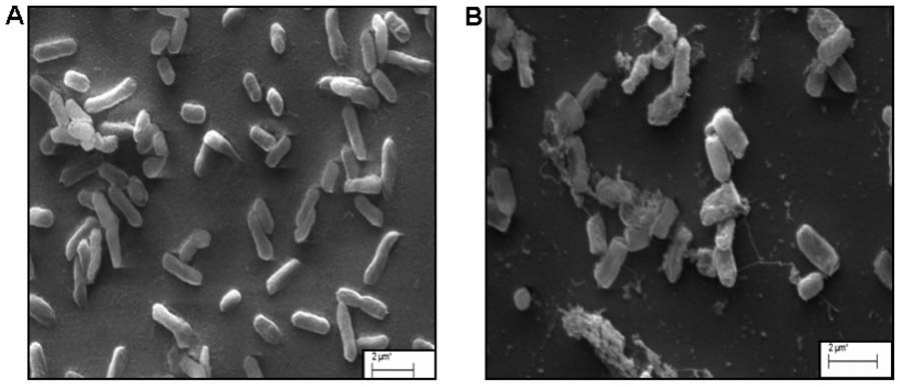
Bactericidal effect of laterosporulin on *E. coli*. The scanning electron micrographs of *E. coli* cells without laterosporulin treatment (A) and after laterosporulin treatment (500 µg/ml) for 4 h (B). Note that the treatment of *E. coli* displaying roughening of cell surface with cell debris as a result of treatment.

This was confirmed by MIC (minimum inhibitory concentration) analysis, where above strains were found to be inhibited at lower MIC values compared to other test strains (**Fig. 4**). To gain insight into the mode of bactericidal action of the laterosporulin on indicator strain, we performed scanning electron microscopy (SEM) of *E. coli* treated with lethal dose of bacteriocin. When compared to untreated bacteria, *E. coli* cells pre-incubated with 500 µg/ml of purified peptide for 4 hours displayed significant modifications in cell shape and morphology (**Fig. 5**). The modifications included roughening of the cell surface with accumulation of cell debris and lysis of bacteria.

### Temperature, pH stability and resistance to proteolytic enzymes

The results of heat stability assay for laterosporulin confirmed that the bacteriocin was fully heat stable as there was no reduction in antimicrobial activity even after exposing it to 120°C for 15 min. It was also found to be stable under a wide range of pH as there was no reduction in antimicrobial activity observed between 2.0 to 10.0. However, the antimicrobial activity of laterosporulin was reduced significantly above pH 10. The antimicrobial assay performed upon incubation of laterosporulin with proteolytic enzymes (pepsin, trypsin, chymotrypsin, proteinase K, pronase E) did not show any reduction in its antimicrobial activity. Further, no reduction in antimicrobial activity upon treatment of laterosporulin with amylase indicates the absence of any sugar moiety associated with antimicrobial activity.

### Analysis of the laterosporulin encoding gene cluster

**Figure 6:**
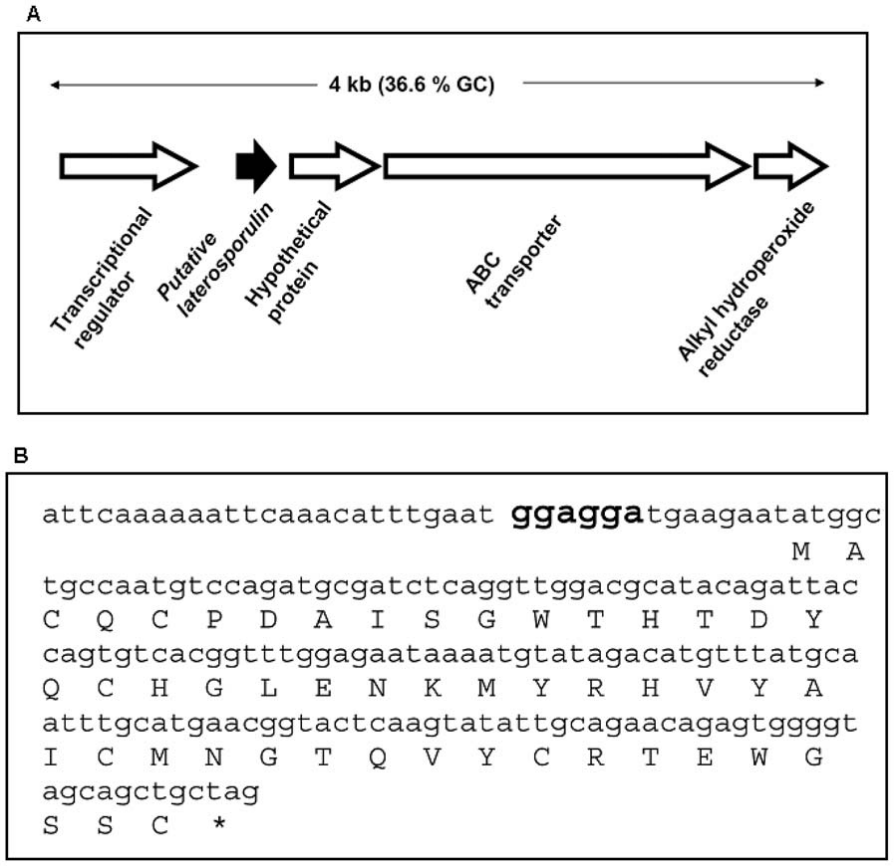
Genetic organization of 4 kb region of the genome encoding laterosporulin (A) and nucleotide sequence of the ORF (B). The putative ORF encoding laterosporulin structural gene is shown by filled arrow and flanking ORFs as shown by empty arrows (A). Panel B shows the nucleotide sequence of the laterosporulin gene (encoding the indicated amino acids) with putative start codan, stop codons and ribosome binding site (RBS) shown in bold.

To identify the ORF that encodes the laterosporulin, we identified a 4 kb region from the preliminary draft genome of GI9 (unpublished). We further confirmed the 4 kb region (HE579167) by re-sequencing (see Methods). Genomic organization of the 4 kb region that contains the putative structural gene encoding laterosporulin and its flanking genes is schematically shown in **Fig. 6A**. The predicted ORF of 153 nucleotides following the putative Shine-Dalgarno sequence (**Fig. 6B**) most likely codes for the antimicrobial peptide. In agreement with this, the deduced amino acid sequence (of the ORF) fully matches with the partial N-terminal sequence of the peptide. However, the homology searches revealed that the ORF predicted is novel to our species and there is no similar or identical homolog has been identified in any other species of bacteria. Recently genome sequence of another strain (LMG 15441) identified as *Brevibacillus laterosporus* has been submitted in NCBI (GenBank accession No AFRV01000005). Like in our strain, all the 4 kb sequence is also present in the genome of this strain and shows 98.3% nucleotide identity. Even though the genomic region is present in this strain, the putative ORF that encodes the laterosporulin is not annotated and missed by their annotation pipeline (although it is encoded in the genome and is 100% identical at nucleotide sequence). The region on the right side of this ORF is flanked by a putative transcriptional regulator gene and on its left side by three putative genes that encode a hypothetical protein, ABC transporter protein and a dehydrogenase gene. The list of predicted ORFs and their most similar homologs is given in **Table S1**. The transcriptional orientation of all ORFs is in the same direction (**Fig. 6a**) and the G+C content of this 4 kb region is only 36.6 mol%, which is somewhat lower than the average G+C content of the genome of *Brevibacillus* sp. strain GI-9 estimated to be 41 mol%.

## Discussion

Members of the genus *Bacillus* are well known to produce bacteriocins, largely classified as lantibiotics with many placed within the class II bacteriocins [15], [19]. Apart from *Bacillus*, genus *Paenibacillus* is also known to produce antimicrobial substances such as polymixins. However, species belonging to other genera like *Brevibacillus* and *Geobacillus* are sparsely explored for antimicrobials [25]–[29]. Thus, exploration of these genera and other close relatives for antimicrobials may result in isolation of novel bacteriocins for various applications, in addition to increasing the knowledge in this field. This study provides the first characterization of a bacteriocin produced by *Brevibacillus* sp. strain GI-9 with activity against a diverse range of bacteria. The most essential parameter for characterization of a bacteriocin is obtaining it in a pure form using different chromatographic techniques [30], [31] including size exclusion and affinity chromatography [32]–[36] after ammonium sulfate precipitation. The purification of laterosporulin involved extraction from a 48 hour culture supernatant using Diaion HP-20 resins, followed by filtration chromatography and reversed-phase high performance liquid chromatography. As an alternative to the ammonium sulfate precipitation few studies reported utilization of hydrophobic interaction of resins to extract selective peptides [20], [37]. Our results clearly suggest that the utilization of Diaion HP-20 could result in high yield and subsequent additional chromatography resulted in obtaining the pure compound.

The purified laterosporulin showed a single band on Tricine-SDS-PAGE, the molecular mass analysis of this peptide using calibration curve obtained from gel filtration chromatography and MALDI-TOF revealed low molecular weight which is generally observed for bacteriocins. The broad elution profile obtained in gel filtration chromatography could be due to conformational polydispersity which is not too uncommon for peptides. To ascertain the molecular mass with higher reliability, we carried out MALDI-TOF of the active peak which gave a mass of 5.6 kDa. In fact, the molecular weight of intact peptide obtained by gene sequence slightly differed with the mass obtained by MALDI-TOF. We speculate that this is a result of peptide maturation where the N-terminal extension of precursor peptide is cleaved off by the action of an enzyme like Met aminopeptidase (MAP) that cleaves at a Met residue when the adjacent residue is a nonbulky amino acid [38]. However, unlike the class II bacteriocins or some of the class I lantibiotics where maturation proteases are associated with an ABC transporter gene [39], strain GI-9 do not contain any significant N-terminal protease domain in ABC transporter. Nonetheless, four copies of genes exhibiting similarity with the MAP gene are found in different locations of the genome. The maturation of peptide is also noticeable from the repeated N-terminal sequences of the peptide which yielded Ala as the first amino acid. The theoretical mass calculated for the peptide obtained from gene sequence upon maturation of peptide (5619.3 Da) is still 13.5 Da higher than the experimental mass observed. However, Gaussian fit to the single peak observed in MALDI-TOF experiment suggested that the peptide molecules have a flight time corresponding to mass of 5605.8 ±33.6 Da and this range covers the theoretical mass of the peptide. Low similarity of the laterosporulin amino acid sequence with existing bacteriocins suggests that it could be a novel bacteriocin. Moreover, the continuous N-terminal sequence obtained in the present study revealed the absence of modified amino acids like dehydrated Ser or Thr which are formed as a result of post-translational modifications. In addition to this, treatment of peptide with dithiothreitol (DTT), used to disrupt disulfide bonds, did not affect laterosporulin's antimicrobial activity or result in any migration differences on SDS-PAGE (data not shown). Further, the bacteriocin is neither composed of two peptides nor contain YGNGVXC motif at N-terminus, which is observed in anti-listerial pediocine like bacteriocins. Interestingly, though laterosporulin did not show the YGNGVXC motif, it could inhibit the growth of test strain *L. monocytogenes*, supporting the hypothesis that YGNGVXC motif does not play any role in inhibiting the growth of *Listeria* [40]. These several lines of evidence suggest that the antimicrobial peptide in the present study belongs to a novel heat stable class II bacteriocin family [41]. In addition to this, the PMF analysis of the antimicrobial peptide using MASCOT and PROWL database analyses revealed no significant similarity with any of the known bacteriocins or antimicrobial peptides produced by bacteria. This was in agreement with the BLAST analysis of a partial N-terminal amino acid sequence against the GenBank protein database and an antimicrobial peptide database that did not reveal any similarity with known antimicrobial peptides. Interestingly, the partial N-terminal sequence of the peptide revealed the absence of cationic amino acids such as Lys or Arg, but it still exhibited antimicrobial activity. Recently, another N-terminal sequence was also reported to lack cations in its partial sequence [20].

One of the hallmarks of the ORFs that encode bacteriocins is their small size (50–70 aa). Due to their small size, genes encoding bacteriocins are often difficult to identify. In our case, the availability of the purified peptide, its partial N-terminal sequence and the draft genome of the source strain helped to identify the putative ORF that encodes the laterosporulin, along with adjacent genes. As expected, the putative ORF that encodes the laterosporulin is only 153 bp (50 aa). The other hallmark of a genomic region that encodes a bacteriocin is the presence of genes necessary for regulation, transport, modification, etc. The 4 kb region encodes such genes and the fact that transcriptional orientation of all these genes is in same direction suggests that they are part of a genetic cassette responsible for laterosporulin production (**Fig. 6A**). Even though bacteriocins are known to be strain specific, the presence of an ORF with significant similarity to the putative laterosporulin-encoding ORF in the genome of *Brevibacillus laterosporus* strain LMG15441 [42] suggests that this strain may produce a related bacteriocin. This highlights the importance of conducting a functional screen to identify bacteriocins.

Many antibiotics are already in use to combat disease or to avoid the food spoilage. Consequently, increased use of these antibiotics resulted in multiple antibiotic resistance in pathogens and food spoiling bacteria. Thus, it is essential to discover novel antimicrobial substances to combat these drug resistant bacteria. The properties such as thermo-stability, pH tolerance and resistance to proteolytic enzymes observed for bacteriocins has fostered their therapeutic and food preservation applications. In this study, laterosporulin was found to be thermo stable, pH tolerant and resistant to proteolytic enzymes. These properties of laterosporulin can increase the potential applications of the bacteriocin alone or in combination with other bacteriocins.

## Materials and Methods

### Isolation and growth media

The bacterial strain GI-9 was isolated from a subsurface farmland soil sample in Chandigarh, located in northern part of India. No specific permissions were required for sample collection as the location is with in the institute's campus and the study did not involve any recombinant materials. The sample was collected and immediately transferred to laboratory for processing. It was serially diluted and plated onto tryptone soya agar (TSA) medium with the following composition (g/l) pancreatic digest of casein, 15.0; papaic digest of soybean meal, 5.0; sodium chloride, 5.0; agar 15.0 and the pH adjusted to 7.2. The plates were incubated for 4 days at 30°C under anaerobic conditions. Colonies with inhibition zones were isolated, purified and preserved at −70°C for further studies. All test strains in the present study were procured from Microbial Type Culture Collection (MTCC), Chandigarh, India and grown on TSA.

### Bacterial identification

Morphological characteristics of cell and spore were observed under phase contrast microscope (Zeiss, Axiophot). Physiological tests like growth at different temperature and pH were determined using TSA as basal medium. Other biochemical tests were performed according to the standard procedures [43], [44]. PCR amplification of the 16 S rRNA gene was done using universal primers 8-27f (5′-AGAGTTTGATCCTGGCTCAG-3′) and 1492r (5′-TACGGYTACCTTGTTACGACTT-3′). Amplified PCR product was purified and sequenced as described [45]. Almost complete sequence of 16 S rRNA gene (submitted to EMBL under the accession No.FR686596) was used for BLAST search using NCBI and EZ Taxon servers.

### Determination of bacteriocin activity

The CFB of strain GI-9 was used to determine the antimicrobial activity. Culture was grown for 48 hours in nutrient broth (NB, Himedia) and subsequently cells were removed by centrifugation (10,000 rpm for 10 min, 4°C). The supernatant obtained was filtered by using 0.22 µm filter (Millipore). The filtrate was diluted by two fold dilution and amounts of 100 µl of different dilutions were used to test the activity. These dilutions were added to the wells that are prepared in plates containing nutrient agar medium seeded with test organism. Above test was also performed using minimal medium to check influence of the medium components on antimicrobial activity. A growth curve up to 24 hours was prepared for strain GI-9 to examine the bacteriocin production at different stages. One unit of bacteriocin was defined as the lowest dilution that was given an inhibition zone around the well and reciprocal of this dilution is defined as the unit of antimicrobial activity per ml.

### Bacteriocin production and purification

An isolated colony was used to inoculate 50 ml of NB medium and incubated at 30°C with shaking at 120 rpm for 24 hours. This culture was used to inoculate 1 liter flasks containing 500 ml of NB. The culture was grown for 48 hours at 30°C and subsequently cells were separated by centrifugation (10,000 g, 15 min at 4°C). The cell free supernatant was mixed with 2% (w/v) of Diaion HP-20 (Supelco) resin and the bacteriocin was eluted with methanol as described [35]. Methanol was evaporated using a rota vapor (BUCHI Rota vapor R-200) and the dried peptide content was dissolved in Milli-Q water. This crude extract was passed through a 30 kDa protein concentrator (Millipore, USA) and the active fraction obtained was applied onto a manually packed and calibrated sephacryl HR-100 column 16/66 (GE Healthcare) linked to an AKTA prime plus (GE Healthcare). The elution was done in 50 mM sodium phosphate buffer (pH 7.2) containing 50 mM NaCl at flow rate of 0.5 ml/min and monitored through UV detector (220 nm). Fractions of 5 ml were precipitated through TCA and re-dissolved in Milli Q water to test the antimicrobial activity. The active fraction was applied onto gel filtration HPLC column (Shodex KW-803) along with molecular weight standards insulin, ribonuclease A, chymotrypsinogen, ovalbumin, conalbumin, ferritin and blue dextran. Finally the bacteriocin was applied onto reversed-phase chromatography (Schimadzu Scientific Instruments, Japan) and purified using semi-preparative C-18 column (Phenomenex, Luna 5μ C-18). The bacteriocin was eluted with 0–100% linear gradient of acetonitrile containing 0.1% trifloroacetic acid (TFA) with a flow rate of 0.5 ml/min. The purified bacteriocin was collected, lyophilized and re-dissolved in water to appropriate concentration. It was used to test antimicrobial activity and applied on Tricine-SDS-PAGE (16.5%).

### Determination of minimum inhibitory concentration

The MIC of bacteriocin for different strains was evaluated by using a microtiter plate dilution assay. Test strains were grown to logarithmic phase under optimal conditions (up to 0.3 OD) and the test was performed in triplicates. Each well of the microtiter plate was added with 200 µl of fresh nutrient medium and 50 µl of test strains in different rows. Subsequently, different dilutions (50 µl) of freshly prepared bacteriocin were added to each well. The first column of the microtiter plate was left as a blank while reading through an ELISA plate reader (Thermo Scientific). The microtiter plates were incubated at 30°C incubator and OD was measured at 600 nm at different time intervals. The lowest concentration that inhibited growth of test strains and did not show any increase in absorption after 48 h was considered as MIC.

To examine the bactericidal activity, *E.coli* cells were grown in NB to an exponential phase, harvested by centrifugation. The pellet obtained was resuspended in fresh NB and aliquots of 5 ml containing about 2×10^7^ cells/ml were incubated at 37°C for 4 hours with 500 µg/ml laterosporulin. Samples at 0, 2 and 4 hours interval were centrifuged and pellets were resuspended in 500 µl of phosphate buffer. Each sample was spread on a poly (L-lysine)-coated glass slides (18×18 mm) to immobilize bacterial cells and incubated at 30°C for 90 min. They were fixed with modified Karnovsky's fixative [46] and dehydrated with a graded ethanol series. After freeze drying and platinum coating, the samples were observed with a Zeiss EVO 40 instrument.

### Effect of pH, temperature and hydrolytic enzymes on bacteriocin activity

The sensitivity of the purified bacteriocin towards different pH, temperature and proteases was evaluated. To determine pH and temperature resistance, the purified peptide was incubated at different pH values between 2.0–12.0 and temperatures 80, 100°C for 30 min and 120°C for 15 min. Different hydrolytic enzymes including pepsin, trypsin, chymotrypsin, proteinase K, pronase E and amylase were incubated with bacteriocin for 6 hours at 37°C to ensure their effect. The enzyme activity was terminated by heating at 80°C before the bacteriocin activity was confirmed.

### Intact mass analysis and peptide mass fingerprinting

For intact mass analysis of bacteriocin, 1 µl of the peptide sample was mixed with equal amount of α-cyano-4-hydroxycinnamic acid in 0.1% (v/v) TFA. Sample was dried and analyzed on an ABI voyager DE STR mass spectrometer (Applied Biosystems). The HPLC purified, functional entity was loaded on to Tricine-SDS-PAGE, upon electrophoresis the protein band was subjected to overnight digestion (at 37°C for about 16 hours) using trypsin and the mass of the generated fragments were analyzed [47]. The observed mass and their relative intensities were used to identify the parent sequence by peptide mass fingerprinting against profiles of known sequences using MASCOT and PROWL servers.

### N-terminal amino acid sequencing and analysis

After separation by Tricine-SDS-PAGE, the peptides were transferred on to PVDF membrane (Bio-Rad), rinsed with Milli-Q water, stained with amido black (Sigma) for 2-3 min and destained with several changes of 50% methanol. The membrane was finally rinsed in Milli-Q water. The excised peptide band was subjected to N-terminal sequencing. The N-terminal sequence was determined by automatic degradation in Procise 491 cLC protein sequencer (Applied Biosystems). The partial sequence obtained was analyzed for its ability to resemble profile of antibacterial peptide by comparing with sequences of peptides with known potency against microbes [48] (http://aps.unmc.edu/AP/main.php).

### Peptide synthesis

To evaluate antibacterial function of the N-terminal peptide, a 19-mer peptide representing the N-terminal sequence of the bioactive protein was synthesized using standard Fmoc-chemistry on 2-chlorotrityl resin (0.2 mmol/g). Synthesis was carried out on a semi-automated synthesizer (PS-II) (Protein Technologies Inc., AZ USA). It was cleaved with TFA and purified by RP-HPLC system (Dionex, Ultimate-3000) using C-18 analytical column. The purified peptide was subjected to MALDI-TOF analysis for confirmation of molecular mass.

### DNA sequence analysis

The DNA was isolated as described by Sambrook et al. [49]. TBLASTN was carried out using the laterosporulin N-terminal sequence against the draft genome of our strain GI-9 (unpublished) to fish out region encoding the putative structural gene for the bacteriocin and its flanking genes. Further, this region was re-sequenced using a set of overlapping primers (**Table S2**) on an ABI 310 Genetic Analyzer (Applied Biosystems) and annotated using NCBI ORF finder (http://www.ncbi.nlm.nih.gov/projects/gorf/). Homology searches were performed at NCBI (http://www.ncbi.nlm.nih.gov/) and published bacteriocin databases such as antimicrobial peptide database, BAGEL and BACTIBASE [50]–[52]. The 4 kb sequence of laterosporulin gene cluster described in the present study has submitted to EMBL under the accession number HE579167.

## Supporting Information

Table S1 Homologs of predicted products of ORFs in the of 4 kb genomic region encoding the putative structural gene for laterosporulin.
(DOC)

Table S2 List of primer for re-sequencing of 4 Kb genomic region encoding the putative structural gene for laterosporulin.
(DOC)
