# Supplementary material for: Identification, Purification and Characterization of Laterosporulin, a Novel Bacteriocin Produced by Brevibacillus sp. Strain GI-9
Source: PLoS One. 2012 Mar 5;7(3):e31498. doi: 10.1371/journal.pone.0031498 (PMC3293901; doi:10.1371/journal.pone.0031498)
Supplement: Table S1 — Homologs of predicted products of ORFs in the of 4 kb genomic region encoding the putative structural gene for laterosporulin. (DOC) [file pone.0031498.s001.doc]

**Table S1:** Homologs of predicted products of ORFs in the of 4 kb genomic region encoding the putative structural gene for laterosporulin.

| **GENE** | **GC CONTENT** | **SIZE** | **REGION** | **Organism** | **RELATED HIT** |
| --- | --- | --- | --- | --- | --- |
| Transcriptional regulator, LuxR family (ORF1) | 33.9% | 693 bp | 35-727 | Hypothetical protein BRLA_c36410 [Brevibacillus laterosporus  LMG 15441],  Identities = 219/222 (99%)[Expect = 1e-162] | Transcriptional regulator, LuxR family [Paenibacillus curdlanolyticus  YK9] Identities = 40/140 (29%)[Expect = 6e-08], ATP-dependent transcriptional regulator, MalT-like, LuxRfamily [Clostridium lentocellum DSM 5427]Identities = 57/227 (25%)[Expect = 1e-07],  Response regulator, positive activator of uhpT transcription [Cytophaga hutchinsonii ATCC 33406] Identities = 26/66(39%) [Expect = 1e-07], transcriptional regulator, LuxR family [Mucilaginibacter paludis  DSM 18603] Identities = 27/64 (42%)[Expect = 1e-07],  transcriptional regulator, LuxR family [Mucilaginibacter paludis  DSM 18603] Identities = 28/64 (44%)[Expect = 2e-07] |
| Putative laterosporulin gne(ORF2) | 43.8% | 153 bp | 982-1134 | None | None |
| Hypothetical protein (ORF3) | 34.8% | 486 bp | 1223-1708 | Hypothetical protein BRLA_c36420 [Brevibacillus laterosporus  LMG 15441],  Identities = 159/161 (99%)[Expect = 2e-114] | Hypothetical protein PPE_00172 [Paenibacillus polymyxa E681] Identities = 159/161 (99%)[Expect = 3e-14], hypothetical protein PaecuDRAFT_2147 [Paenibacillus curdlanolyticus  YK9] Identities = 42/153 (27%)[Expect = 2e-11], Hypothetical protein [Bacillus halodurans C-125]  Identities = 26/92 (28%)[Expect = 0.10], Thioredoxin domain-containing protein [Anoxybacillus flavithermus  WK1]Identities = 31/123 (25%)[Expect = 0.49  ], conserved domain protein [Paenibacillus sp. HGF7] Identities = 29/133 (22%)[ Expect=0.60], Serine/threonine-protein kinase TNNI3K isoform 1 [Macaca mulatta]Identities = 21/52 (40%)[Expect = 6.8] |
| ABC transporter(ORF4) | 38.8% | 1851 bp | 1711-3561 | Subtilin transport ATP-binding protein SpaT [Brevibacillus laterosporus  LMG 15441]  Identities = 581/593 (98%)[ Expect = 0.0] | ABC transporter related protein [Paenibacillus curdlanolyticus  YK9] Identities = 205/585 (35%)[Expect = 4e-123], ABC transporter, ATP-binding protein [Paenibacillus sp. HGF7] Identities = 201/576 (35%)[Expect = 5e-120], Multidrug ABC transporter protein [Paenibacillus polymyxa E681] ABC superfamily ATP binding cassette transporter Identities = 198/575 (34%)[ Expect = 2e-112], ABC superfamily ATP binding cassette transporter, membrane protein  [Bacillus cereus F65185] Identities = 159/510 (31%)[ Expect = 5e-75], ABC transporter ATP binding/permease protein [Brevibacillus brevis  NBRC 100599] Identities = 175/604 (29%)[Expect = 3e-73] |
| Alkyl hydroperoxide reductase (ORF5) | 38.6% | 435 bp | 3566-4000 | Alkyl hydroperoxide reductase/ thiol specific antioxidant/ Mal  allergen [Brevibacillus laterosporus LMG 15441]  Identities = 140/144 (97%)[ Expect = 9e-99] | Alkyl hydroperoxide reductase/ Thiol specific antioxidant/ Mal  Allergen [Paenibacillus curdlanolyticus YK9] Identities = 55/136 (40%)[ Expect = 2e-39]  , Methylamine utilization protein [Paenibacillus polymyxa E681] Identities = 53/124 (43%)[ Expect = 7e-27], conserved domain protein [Paenibacillus sp. HGF7] Identities = 51/138 (37%)[ Expect = 2e-25], hypothetical protein ANT_05500 [Anaerolinea thermophila UNI-1] Identities = 41/124 (33%)[ Expect = 3e-12], hypothetical protein Mpe_A2654 [Methylibium petroleiphilum PM1]  [Expect = 2e-08],methylamine dehydrogenase accessory protein MauD [Methylotenera  mobilis JLW8]  Identities = 39/140 (28%)[Expect = 7e-08], methylamine dehydrogenase accessory protein MauD [Achromobacter  xylosoxidans C54] Identities = 40/139 (29%)[Expect = 3e-06] |
