# Supplementary material for: Identification, Purification and Characterization of Laterosporulin, a Novel Bacteriocin Produced by Brevibacillus sp. Strain GI-9
Source: PLoS One. 2012 Mar 5;7(3):e31498. doi: 10.1371/journal.pone.0031498 (PMC3293901; doi:10.1371/journal.pone.0031498)
Supplement: Table S2 — List of primer for re-sequencing of 4 Kb genomic region encoding the putative structural gene for laterosporulin. (DOC) [file pone.0031498.s002.doc]

**Table S2:** List of primer for re-sequencing of 4Kb genomic region encoding the putative structural gene for laterosporulin.

| **Name** | Sequence |
| --- | --- |
| LTSF1 | ttcctgttgcgaccagacat |
| LTSF2 | taccaacaaggatgcgaacagcat |
| LTSF3 | tttcgaagtggttcgcaggtc |
| LTSF4 | gtgtaggccaaattatcacttgcggt |
| LTSF5 | ggggaatttccatttatgtgctggt |
| LTSF6 | gcagctggaaaatctacgctg |
| LTSF7 | aggtacaaccattccaccca |
| LTSR1 | ggatcgtttccattgagaacgcta |
| LTSR2 | tctggacattggcaagccat |
| LTSR3 | agacatccagaaacggttcca |
| LTSR4 | ggttatgagcccgcgccatttgta |
| LTSR5 | cctgaaagattgccccacag |
| LTSR6 | cttatcgatccaaggtggggt |
| LTSR7 | gcaatttcgcaggatggcaa |
